# Dietary patterns and accelerated multimorbidity in older adults

**DOI:** 10.1038/s43587-025-00929-8

**Published:** 2025-07-28

**Authors:** David Abbad-Gomez, Adrián Carballo-Casla, Giorgi Beridze, Esther Lopez-Garcia, Fernando Rodríguez-Artalejo, Maria Sala, Mercè Comas, Davide Liborio Vetrano, Amaia Calderón-Larrañaga

**Affiliations:** 1 Department of Epidemiology and Evaluation, Hospital del Mar, Barcelona, Spain; 2 Hospital del Mar Research Institute, Barcelona, Spain; 3 Research Network on Chronicity, Primary Care and Health Promotion (RICAPPS), Instituto de Salud Carlos III (ISCIII), Madrid, Spain; 4 Aging Research Center, Department of Neurobiology, Care Sciences and Society, Karolinska Institutet and Stockholm University, Stockholm, Sweden; 5 PhD Program in Biomedicine, Universitat Pompeu Fabra, Barcelona, Spain; 6 Center for Networked Biomedical Research in Epidemiology and Public Health (CIBERESP), Madrid, Spain; 7 Department of Preventive Medicine and Public Health, Universidad Autónoma de Madrid, Madrid, Spain; 8 IMDEA Food Institute, Campus of International Excellence, Madrid, Spain; 9 Stockholm Gerontology Research Center, Stockholm, Sweden

**Keywords:** Diseases, Medical research, Ageing

## Abstract

Diet could influence disease development and shape multimorbidity trajectories. Here we examined how four dietary patterns relate to 15-year multimorbidity accumulation in 2,473 community-dwelling older adults from the Swedish SNAC-K cohort. Multimorbidity was operationalized as the total number of chronic conditions and grouped into three organ systems. Higher adherence to the Mediterranean-DASH Diet Intervention for Neurodegenerative Delay, the Alternative Healthy Eating Index and the Alternative Mediterranean Diet was inversely associated with the annual rate of total chronic disease accumulation (β coefficient (95% confidence interval) per 1-s.d. increment: −0.049 (−0.065 to −0.032), −0.051 (−0.068 to −0.035) and −0.031 (−0.048 to −0.014), respectively), whereas higher adherence to the Empirical Dietary Inflammatory Index was associated with a faster rate of accumulation (0.053 (0.035–0.071)). Similar associations were observed for cardiovascular and neuropsychiatric diseases but not for musculoskeletal diseases. Some associations varied by sex and age. Our findings support diet quality as a modifiable risk factor for multimorbidity progression in older adults, with possible implications for dietary guidelines, public health strategies and clinical practice.

## Abstract (web summary)

In a Swedish cohort, Abbad-Gomez et al. report that diet quality is inversely associated with the rate of accumulation of total, cardiovascular and neuropsychiatric chronic diseases in older adults, supporting diet quality as a modifiable risk factor for multimorbidity progression.

## Main

Aging is one of the main risk factors for several chronic diseases, and it has, thus, been associated with higher morbidity burden, especially with neurodegenerative^1^ and cardiovascular conditions^2^. In 2021, the burden of disease in high-income countries, measured as disability-adjusted life years, was almost five times higher among people older than 70 years compared to people aged 20–54 years^3^. Accordingly, one of the main objectives of the World Health Organization when promoting healthy aging is to enable older persons to live longer, healthier lives according to their values and preferences, even while living with chronic diseases^4,5^.

Multimorbidity—that is, the presence of two or more concurrent chronic diseases^6^—is becoming an increasingly prevalent and relevant phenomenon within the study of burden of disease, as it focuses more on the persons affected by diseases and less on the diseases themselves. Although there is no consensus on multimorbidity measurement or operationalization^7^, its clustering in cardiovascular, neuropsychiatric and musculoskeletal organ systems has been a widely acknowledged way to operationalize multimorbidity burden^8–11^.

Among the factors known to influence the risk of chronic conditions, diet and nutrition are at the center^12–14^. Different dietary patterns (that is, combinations of foods and nutrients) have shown protective associations with various health outcomes, including cardiovascular disease, cancer, type 2 diabetes mellitus, cognitive decline and dementia^15,16^. Assessing diet quality with dietary patterns instead of individual foods or nutrients can account for the cumulative effects of dietary components and possible interactions between them, which could reveal associations with health outcomes that might otherwise remain hidden^17,18^.

The few studies that have explored the relationship between diet and multimorbidity have found opposite associations of healthy and unhealthy dietary patterns (for example, Alternate Healthy Eating Index (AHEI) and Western diet) with multimorbidity onset^19,20^. Nevertheless, most of these studies have focused on index diseases and their comorbidities, single dietary patterns, foods or nutrients and/or relied on short follow-up times and/or cross-sectional assessments of both diet and diseases^21–26^.

Accordingly, the main objective of the present study was to explore the long-term speed of chronic disease accumulation in relation to the cumulative adherence to multiple dietary patterns previously linked to individual cardiovascular, neuropsychiatric and musculoskeletal chronic diseases, namely the Mediterranean-DASH (Dietary Approaches to Stop Hypertension) Diet Intervention for Neurodegenerative Delay (MIND)^27^, the AHEI^28^, the Alternative Mediterranean Diet (AMED)^29^ and the Empirical Dietary Inflammatory Index (EDII)^30^. The simultaneous study of conceptually different dietary patterns in a population of older adults can provide insights on whether several diets could confer similar benefits on multimorbidity^31^.

## Results

### Descriptive results

**Extended Data Fig. 1:**
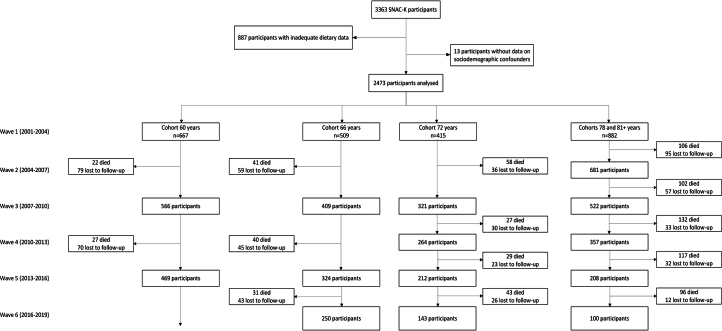
**Flow chart of the study participants**.

Characteristics of the study population are shown in Table 1. Mean age at baseline was 71.5 years (s.d. = 9.39); 61.1% of the study participants were females; and 84.3% had multimorbidity. Most participants were followed at least once or twice (80.1% and 63.8%, respectively) over the 15-year study period (Extended Data Fig. 1).

**Table 1:** Baseline adherence to the dietary patterns, by socioeconomic, lifestyle and morbidity characteristics of the study population

		MIND	AHEI	AMED	EDII
	*N* = 2,473	Range (2–12)	Range (26.15–86.98)	Range (0–9)	Range (−1.36 to 2.70)
	*n* (%)	Mean (s.d.)	Mean (s.d.)	Mean (s.d.)	Mean (s.d.)
Age					
60 to <65 years	667 (26.97%)	7.15 (1.73)	63.70 (9.29)	4.55 (1.72)	−0.03 (0.30)
65 to <70 years	509 (20.58%)	6.91 (1.82)	62.15 (10.09)	4.37 (1.84)	0.04 (0.31)
70 to <80 years	793 (32.07%)	6.68 (1.64)	61.48 (9.25)	4.32 (1.74)	0.07 (0.28)
≥80 years	504 (20.38%)	6.14 (1.63)	57.23 (9.88)	3.73 (1.64)	0.13 (0.31)
Sex					
Male	961 (38.86%)	6.47 (1.74)	59.56 (9.79)	4.39 (1.70)	0.05 (0.31)
Female	1,512 (61.14%)	6.92 (1.71)	62.49 (9.68)	4.19 (1.79)	0.05 (0.29)
Living arrangement					
Alone	1,250 (50.55%)	6.72 (1.76)	60.39 (10.16)	4.03 (1.78)	0.06 (0.31)
Not alone	1,223 (49.45%)	6.77 (1.71)	62.33 (9.38)	4.52 (1.70)	0.03 (0.29)
Previous occupation					
Manual worker	486 (19.65%)	6.41 (1.71)	59.62 (9.96)	4.09 (1.69)	0.11 (0.34)
Non-manual worker	1,987 (80.35%)	6.82 (1.73)	61.77 (9.75)	4.31 (1.77)	0.03 (0.29)
Education					
Elementary	319 (12.90%)	6.18 (1.73)	57.78 (10.28)	3.91 (1.75)	0.14 (0.33)
High school	1,223 (49.45%)	6.65 (1.67)	60.98 (9.36)	4.11 (1.73)	0.06 (0.29)
University	931 (37.65%)	7.06 (1.76)	63.06 (9.90)	4.60 (1.75)	−0.00 (0.29)
Tobacco smoking					
Never	1,089 (44.04%)	6.75 (1.73)	61.49 (9.53)	4.31 (1.76)	0.08 (0.29)
Former smoker	987 (39.91%)	6.84 (1.71)	62.17 (9.65)	4.38 (1.76)	0.03 (0.28)
Current smoker	360 (14.56%)	6.51 (1.79)	59.02 (10.52)	3.88 (1.79)	−0.01 (0.35)
Unknown	37 (1.50%)	6.31 (1.91)	57.89 (12.06)	4.19 (1.70)	0.27 (0.34)
Physical activity					
Inadequate	436 (17.63%)	6.33 (1.77)	57.91 (9.77)	3.68 (1.71)	0.06 (0.28)
Health-enhancing	1,253 (50.67%)	6.70 (1.69)	61.65 (9.66)	4.30 (1.72)	0.05 (0.31)
Fitness-enhancing	619 (25.03%)	7.26 (1.66)	64.01 (9.23)	4.74 (1.66)	0.01 (0.29)
Unknown	165 (6.67%)	6.25 (1.80)	58.18 (9.96)	3.85 (1.85)	0.12 (0.34)
Energy intake					
<1,500 kcal d^−1^	622 (25.15%)	6.88 (1.71)	61.89 (9.32)	3.46 (1.59)	−0.02 (0.22)
1,500–2,000 kcal d^−1^	782 (31.62%)	6.81 (1.71)	62.46 (9.64)	4.11 (1.68)	0.00 (0.24)
>2,000 kcal d^−1^	1,069 (43.23%)	6.61 (1.76)	60.22 (10.13)	4.86 (1.69)	0.12 (0.36)
Chronic diseases (number)					
0–1	339 (13.71%)	7.25 (1.71)	64.51 (9.15)	4.69 (1.62)	−0.05 (0.26)
2–3	908 (36.72%)	6.85 (1.75)	62.02 (9.69)	4.32 (1.76)	0.02 (0.30)
≥4	1,226 (49.58%)	6.53 (1.70)	59.98 (9.86)	4.12 (1.77)	0.09 (0.30)

As shown in Table 1, baseline diet quality decreased as age, number of chronic diseases and energy intake increased. Diet quality was also generally lower among those with lower educational level, those living alone, manual workers, current smokers and the less physically active.

### Main results

Table 2 shows the β coefficients for excess annual change in disease accumulation (95% confidence intervals (CIs)) per 1-s.d. increment in cumulative adherence to every dietary pattern. Differences between the minimally adjusted (Model 1) and fully adjusted models were small. In Model 3, whereas MIND (−0.049 (−0.065 to −0.032)), AHEI (−0.051 (−0.068 to −0.035)) and AMED (−0.031 (−0.048 to −0.014)) showed inverse associations with the rate of total chronic disease accumulation, higher EDII scores were associated with a faster rate of accumulation (0.053 (0.035–0.071)). Similar relationships were observed for the accumulation of cardiovascular diseases (β (95% CI) for MIND, AHEI, AMED and EDII were −0.015 (−0.021 to −0.009), −0.010 (−0.016 to −0.005), −0.005 (−0.010 to 0.000) and 0.013 (0.007–0.019), respectively) as well as for neuropsychiatric diseases (−0.008 (−0.013 to −0.003), −0.012 (−0.016 to −0.007), −0.007 (−0.012 to −0.003) and 0.012 (0.007–0.017), respectively). No associations were found between dietary patterns and the speed of accumulation of musculoskeletal diseases.

**Table 2:** Association between the cumulative adherence to dietary patterns (per 1-s.d. increment) and the yearly rate of disease accumulation during a 15-year follow-up (*N* = 2,473)

		Total chronic diseases (*n* = 60)		Cardiovascular diseases (*n* = 8)		Neuropsychiatric diseases (*n* = 11)		Musculoskeletal diseases (*n* = 5)	
		β (95% CI)	*P* value	β (95% CI)	*P* value	β (95% CI)	*P* value	β (95% CI)	*P* value
MIND	Model 1	−0.050 (−0.067 to −0.033)	<0.001	−0.015 (−0.021 to −0.010)	<0.001	−0.008 (−0.013 to −0.003)	<0.001	−0.002 (−0.007, 0.002)	0.355
	Model 2	−0.050 (−0.067 to −0.033)	<0.001	−0.015 (−0.021 to −0.010)	<0.001	−0.008 (−0.013 to −0.003)	<0.001	−0.002 (−0.007, 0.002)	0.356
	Model 3	−0.049 (−0.065 to −0.032)	<0.001	−0.015 (−0.021 to −0.009)	<0.001	−0.008 (−0.013 to −0.003)	<0.001	−0.002 (−0.006, 0.003)	0.393
AHEI	Model 1	−0.052 (−0.068 to −0.035)	<0.001	−0.010 (−0.016 to −0.005)	<0.001	−0.011 (−0.016 to −0.007)	<0.001	−0.000 (−0.005, 0.004)	0.928
	Model 2	−0.052 (−0.068 to −0.035)	<0.001	−0.010 (−0.016 to −0.005)	<0.001	−0.011 (−0.016 to −0.007)	<0.001	−0.000 (−0.005, 0.004)	0.937
	Model 3	−0.051 (−0.068 to −0.035)	<0.001	−0.010 (−0.016 to −0.005)	<0.001	−0.012 (−0.016 to −0.007)	<0.001	−0.000 (−0.005, 0.004)	0.934
AMED	Model 1	−0.031 (−0.049 to −0.014)	<0.001	−0.005 (−0.011, 0.000)	0.072	−0.007 (−0.012 to −0.003)	0.003	−0.001 (−0.006, 0.003)	0.619
	Model 2	−0.031 (−0.049 to −0.014)	<0.001	−0.005 (−0.011, 0.000)	0.072	−0.007 (−0.012 to −0.003)	0.003	−0.001 (−0.006, 0.003)	0.620
	Model 3	−0.031 (−0.048 to −0.014)	<0.001	−0.005 (−0.010, 0.000)	0.073	−0.007 (−0.012 to −0.003)	0.002	−0.001 (−0.004, 0.005)	0.624
EDII	Model1	0.054 (0.036–0.072)	<0.001	0.013 (0.007–0.019)	<0.001	0.012 (0.007–0.016)	<0.001	0.001 (−0.004, 0.006)	0.706
	Model2	0.054 (0.036–0.072)	<0.001	0.013 (0.007–0.019)	<0.001	0.011 (0.007–0.016)	<0.001	0.001 (−0.004, 0.006)	0.719
	Model3	0.053 (0.035–0.071)	<0.001	0.013 (0.007–0.019)	<0.001	0.012 (0.007–0.017)	<0.001	0.001 (−0.004, 0.005)	0.767

MIND: baseline range: 2–12; 1 s.d. = 1.74

AHEI: baseline range: 29.9–91.7; 1 s.d. = 9.82

AMED: baseline range: 0–9; 1 s.d. = 1.76

EDII: baseline range: −1.36 to 2.70; 1 s.d. = 0.30

Model 1: Linear mixed model with random intercept and slope, adjusted by age (years) and sex (male or female). Models on disease accumulation within a given organ system were adjusted for diseases not belonging to said organ system.

Model 2: As Model 1, additionally adjusted by living arrangements (alone or not), previous occupation (manual or non-manual worker) and education (elementary, high school or university).

Model 3: As Model 2, additionally adjusted by tobacco smoking (never, former smoker, current smoker or unknown), physical activity (inadequate, health-enhancing, fitness-enhancing or unknown) and energy intake (kcal d^−1^).

Two-sided *P* values were obtained from Wald tests. No adjustments were made for multiple comparisons.

**Fig. 1:**
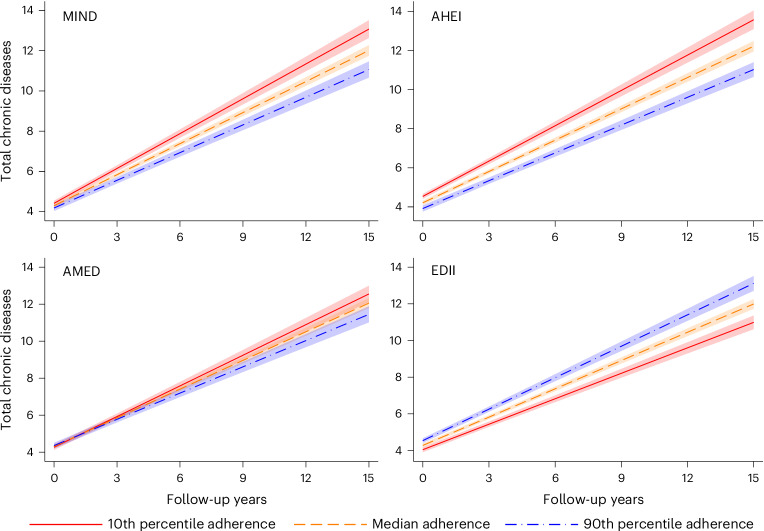
Association between the cumulative adherence to dietary patterns and the yearly rate of total chronic disease accumulation during a 15-year follow-up (*N* = 2,473). MIND: baseline range: 2–12; 1 s.d. = 1.74. AHEI: baseline range: 29.9–91.7; 1 s.d. = 9.82. AMED: baseline range: 0–9; 1 s.d. = 1.76. EDII: baseline range: −1.36 to 2.70; 1 s.d. = 0.30. Model: linear mixed model with random intercept and slope, adjusted by age (years), sex (male or female), living arrangements (alone or not), previous occupation (manual or non-manual worker), education (elementary, high school or university), tobacco smoking (never, former smoker, current smoker or unknown), physical activity (inadequate, health-enhancing, fitness-enhancing or unknown) and energy intake (kcal d^−1^). Data are presented as average predicted number of chronic diseases ± 95% CIs (shaded area).

The associations between cumulative adherence to the dietary patterns and accumulation of total chronic diseases are also plotted in Fig. 1. After the completion of the 15-year follow-up, the difference (95% CI) in the number of chronic diseases between the 10th and 90th percentiles of adherence to the MIND, AHEI and AMED was 2.01 (1.17–2.86), 2.54 (1.70–3.39) and 1.10 (0.23–1.98), respectively. The corresponding difference between the 90th and 10th percentiles of the EDII was 2.13 (1.35–2.90) chronic diseases. The total number of chronic diseases at the 3-year, 6-year, 9-year, 12-year and 15-year follow-ups by levels of adherence to the dietary patterns is shown in Supplementary Table 1.

**Fig. 2:**
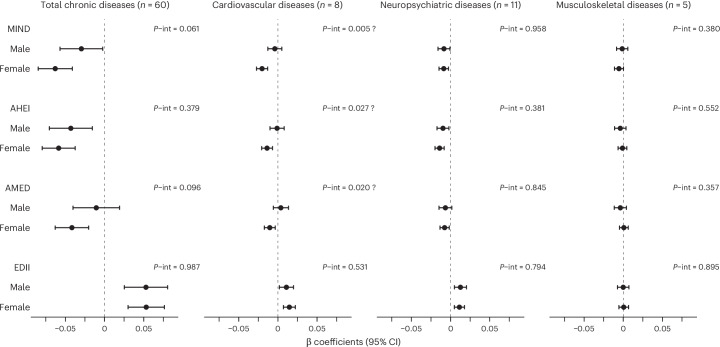
Association between the cumulative adherence to dietary patterns and the yearly rate of chronic disease accumulation during a 15-year follow-up, stratified by sex (male, *n* = 931; female, *n* = 1,512). MIND: baseline range: 2–12; 1 s.d. = 1.74. AHEI: baseline range: 29.9–91.7; 1 s.d. = 9.82. AMED: baseline range: 0–9; 1 s.d. = 1.76. EDII: baseline range: −1.36 to 2.70; 1 s.d. = 0.30. Model: linear mixed model with random intercept and slope, adjusted by age (years), living arrangements (alone or not), previous occupation (manual or non-manual worker), education (elementary, high school or university), tobacco smoking (never, former smoker, current smoker or unknown), physical activity (inadequate, health-enhancing, fitness-enhancing or unknown) and energy intake (kcal d^−1^). Models on disease accumulation within a given organ system were adjusted for diseases not belonging to said organ system. Estimates were obtained from models with multiplicative interaction terms among the dietary patterns, time from inception and sex. Two-sided *P* values for interaction were obtained from Wald tests. ‘?’ indicates not significant when adjusting for multiple comparisons (FDR of 5%). Data are presented as β coefficients for excess annual change in disease accumulation per 1-s.d. increment in cumulative adherence to every dietary pattern ± 95% CIs. int, interaction.

The associations between cumulative adherence to the healthy dietary patterns (MIND, AHEI and AMED) and cardiovascular chronic disease accumulation were apparent only in females, whereas the corresponding associations in males were null (Fig. 2 and Supplementary Table 2). *P* values for interaction were 0.005, 0.027 and 0.020, respectively, but none remained significant when adjusting for multiple comparisons using a false discovery rate (FDR) of 5%. Associations between the EDII and accumulation of total and cardiovascular chronic diseases were similar in males and females. No interactions with sex were found between any dietary pattern and the accumulation of total, neuropsychiatric or musculoskeletal diseases. Stratified associations by age are shown in Supplementary Table 3. The association between the MIND and total chronic disease accumulation was found only in the oldest old (≥78 years; *P* value for interaction was 0.034), similar to those between the MIND and AHEI and neuropsychiatric diseases (*P* values for interaction were 0.036 and 0.047), but no interaction remained significant when using an FDR of 5%. Associations of the AMED and EDII were similar in the youngest and oldest old. No interactions with age were observed between any dietary pattern and cardiovascular or musculoskeletal diseases.

**Extended Data Fig. 2:**
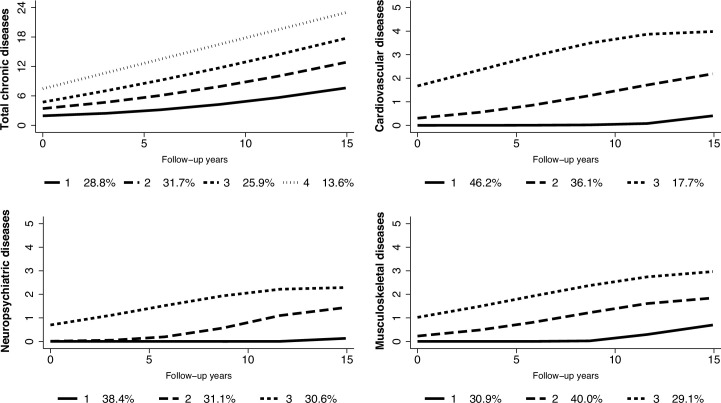
Trajectories of chronic disease accumulation during a 15-year follow-up, adjusted for age and sex (N = 2,473), obtained from group-based trajectory modelling. Percentages indicate the proportion of participants assigned to a given trajectory of chronic disease accumulation.

Associations between dietary patterns and disease accumulation trajectories are displayed in Supplementary Table 4, and the trajectories identified through group-based modeling are plotted in Extended Data Fig. 2. Four trajectories were identified for the total number of chronic diseases and three for cardiovascular, neuropsychiatric and musculoskeletal diseases. Higher average adherence to the MIND (log-odds (95% CI) = −0.185 (−0.345 to −0.025)) and AHEI (−0.188 (−0.345 to −0.032)) were associated with a lower probability of belonging to the second fastest versus the slowest trajectory of total chronic disease accumulation. Opposite results were found for the EDII (0.322 (0.154–0.490)). Higher adherence to the EDII was also associated with higher probability of belonging to the fastest trajectory of cardiovascular chronic disease accumulation (0.216 (0.052–0.381)), whereas higher adherence to the AHEI and AMED was associated with lower probability of belonging to the fastest trajectory of neuropsychiatric disease accumulation (−0.154 (−0.271 to −0.037) and −0.177 (−0.310 to −0.044), respectively). Higher adherence to the AMED was associated with a higher probability of belonging to the fastest trajectory of musculoskeletal disease accumulation (0.150 (0.011–0.288)).

### Ancillary analyses

Adherence to the MIND and AHEI was also inversely associated with the total number of chronic diseases at baseline, whereas the EDII was directly associated with this outcome (Supplementary Table 5). In sensitivity analyses (Supplementary Table 6), restricting the analyses to participants without multimorbidity at baseline substantially reduced the sample size (*n* = 959) and the strength of associations. Specifically, those between the MIND and AMED and total chronic disease accumulation lost statistical significance, although trends remained similar to the main analyses. Using only baseline dietary data also reduced the strength of associations, but they maintained statistical significance. Using only participants who were followed up at least once or had complete case observations, excluding dietary misreporters or observations with probable cognitive impairment, additionally adjusting the analyses for sugar-sweetened beverage consumption and not considering potential cardiovascular risk factors, sleep disorders and genetic conditions in the chronic disease count rendered similar results to the main analyses. The MIND, AHEI and AMED (but not the EDII) were associated with a lower rate of increase in the Royal College of Surgeons Charlson score. Assessing diet quality with the Global Diet Quality Score (GDQS) and the Mediterranean Diet Score (MDS) rendered similar associations to those of the AHEI and AMED, respectively (Supplementary Table 7).

## Discussion

In this cohort of older adults, cumulative adherence to four conceptually different dietary patterns showed strong associations with the speed of total, cardiovascular and neuropsychiatric multimorbidity accumulation over 15 years. Higher adherence to the healthy dietary patterns (that is, MIND, AHEI and AMED) was associated with slower chronic disease accumulation, contrary to what was observed for the inflammatory potential of diet (EDII). Several associations with accumulation of cardiovascular and neuropsychiatric diseases were stronger in females and the oldest old, respectively. Results were consistent in sensitivity analyses as well as when modeling multimorbidity as trajectories of disease accumulation.

### Interpretation

Similar associations between dietary patterns and multimorbidity were found in a previous observational study with a similar operationalization of multimorbidity^19^. Specifically, higher adherence to the AHEI was associated with slower multimorbidity accumulation, whereas the Mediterranean diet showed a non-significant trend. Similar associations with diet quality were also found in studies assessing the multimorbidity of cancer and cardiometabolic diseases^32,33^ and in a study using the Charlson Comorbidity Index^24^. Specifically, higher consumption of ultra-processed foods appears to increase the risk of cancer and cardiometabolic multimorbidity^19,32^, whereas fish/seafood has been associated with lower risk of the latter^33^. Moreover, energy intake, protein intake and vitamin B12 and vitamin D intake have been associated with increased risk of multimorbidity, contrary to vitamin C and iron intake^21,24^.

A potential explanation for our findings relates to inflammation, as all dietary patterns assessed in this study were previously associated with plasma inflammatory markers. Specifically, the MIND, AHEI and AMED have been associated with lower levels of interleukin-6 and C-reactive protein, contrary to the EDII, which has been associated with higher concentrations of these proteins^30,34^. In turn, inflammatory markers have been linked to cardiovascular and neuropsychiatric diseases, which could help explain the higher yearly multimorbidity accumulation associated with lower diet quality in our study, as many older individuals develop inflammaging over time^35^.

In our analyses, the MIND dietary pattern, which was developed as a strategy to preserve brain function during aging^36^, showed protective associations not only with neuropsychiatric multimorbidity accumulation but also with that of cardiovascular multimorbidity and total chronic diseases. This is in line with evidence indicating that the MIND is associated with an improvement in cardiometabolic outcomes such as blood pressure, glycemic control, lipid profile and a reduction of stroke risk^34^. Interestingly, a recent randomized controlled trial in older adults reported a lack of effect of the MIND on 3-year changes in cognition and neuroimaging outcomes, possibly implying that longer-term adherence to this dietary pattern and/or a longer follow-up are needed for potential effects to be observed^27^.

Previous studies also found significant associations between the AHEI and lower risk of individual cardiovascular diseases^37^ and specific neuropsychiatric diseases such as Parkinson’s disease^38^, dementia^39^ or depression^40^. Similar inverse associations were found between the AMED and Parkinson’s disease^38^ and other neurological diseases^41^, even if results regarding specific cognitive domains have been mixed^42^. It is of note that, in our study, associations of the AHEI with multimorbidity were stronger than those found for the other dietary patterns, especially the AMED (which was not significantly associated with cardiometabolic multimorbidity). On the one hand, the former pattern was designed using the foods and nutrients most predictive of chronic disease risk^28^. On the other hand, the AMED may not capture all the components of the Mediterranean diet (for example, olive oil consumption), as it was designed for non-Mediterranean populations, such as the United States^29^. As for our finding of higher adherence to the EDII being related to faster multimorbidity accumulation, a previous study observed associations of the EDII with higher incidence of total cardiovascular disease, coronary heart disease and stroke^43^. This dietary pattern has also been associated with neuropsychiatric diseases such as depression or anxiety but not dementia or Parkinson’s disease^22^.

We found a robust link between most dietary patterns and cardiovascular and neuropsychiatric multimorbidity, but no associations were observed with musculoskeletal diseases. Although there is some evidence regarding the impact of diet on inflammatory arthropathies, osteoarthritis and pathological fractures^44,45^, our null findings are similar to other investigations conducted in the Swedish National study on Aging and Care in Kungsholmen (SNAC-K) cohort, where musculoskeletal multimorbidity was not associated with transitions across cognitive stages and death, incident physical frailty or risk of institutionalization^46–48^. This suggests that at least part of the differences between our results and those of other studies may be population specific and possibly that the association with inflammatory musculoskeletal diseases found in previous studies may become diluted when studying the total musculoskeletal multimorbidity count.

Regarding interaction analyses, we observed that the association of healthy dietary patterns with cardiovascular multimorbidity accumulation was modified by sex. Although there is mixed evidence of the influence of sex on the impact of diet on health, some studies have shown that the Healthy Eating Index is more strongly associated with lower cardiovascular mortality in females than males and that the Nordic dietary pattern is associated with improved muscle strength and physical performance only in females^23,49^. Possible explanations include higher leptin levels in females (and, hence, earlier satiation at equal food consumption) and differences in gut microbiota (which could impact the absorption, distribution and metabolism of nutrients and phytochemicals)^49^; longer life expectancy among women, which could allow for a longer follow-up duration (often needed for an effect of diet on health to be observed); response bias (older women may better report dietary information, as they were more often in charge of household chores); and differences in food choices between males and females. Indeed, equal dietary pattern scores can be obtained from substantially different food combinations, which, in turn, may have different associations with multimorbidity^23,49^.

Finally, the inverse association of the MIND with multimorbidity accumulation was stronger in the oldest old, mostly driven by a slower accumulation of neuropsychiatric diseases that was also observed for the AHEI. This is not entirely unexpected, given that the incidence of diseases such as dementia, Parkinson’s disease and depression/mood disorders largely increases with age (and, hence, statistical power)^50^. An implication of these findings is that behavioral risk factors such as diet may still be targeted in persons well into old age to reduce their risk of developing such highly disabling conditions, for which disease-modifying treatments are often lacking.

### Strengths and limitations

The main strength of this study is the long follow-up time (that is, 15 years) and the repeated dietary assessments over the follow-up, given that the effects of diet on chronic diseases may be cumulative and have long induction periods, and dietary habits can change over time. Furthermore, our analytical approach assessed the relationship of diet with the speed of chronic disease accumulation, which has been deemed a proxy for accelerated aging^51,52^. Finally, we conducted several sensitivity analyses that rendered similar results, including alternative operationalizations of the outcome, increasing the internal validity of study findings.

A limitation of this study is the lack of dietary information before the start of the observation period and the fact that dietary information was self-reported. Specifically, the former limitation prevented us from determining the influence of lifelong exposure to dietary patterns. In addition, the analysis of cumulative dietary behaviors could introduce some reverse causality, as participants could have changed their diet because of their morbidity burden. However, this hypothesis may not be likely, as modeling baseline adherence to the dietary patterns did not substantially change the study associations, and the follow-up was long term. Other ancillary analyses also reduced the risk of reverse causality, as the association trends between diet and multimorbidity persisted when restricting the analyses to participants free from multimorbidity and when using trajectory-based analyses.

Lastly, the SNAC-K comprises urban, mostly community-dwelling, highly educated and relatively affluent Swedish older adults. These characteristics are associated with both higher overall diet quality^53^ and lower risk of multimorbidity^54^, limiting the generalizability of our findings to other populations and settings. Attrition due to dropout and death over the follow-up could have reduced the precision and strength of estimates, even though only one-fifth of study participants were never followed. It is also worth noting that the many subgroup analyses may have led to false-positive interaction terms. Specifically, when using an FDR of 5%, none of the observed interactions with sex and age remained significant.

### Conclusions

In this population-based cohort study, healthy dietary patterns such as the MIND, AHEI and AMED showed a protective association with the speed of accumulation of chronic diseases in older adults over time, whereas the inflammatory potential of diet (EDII) had a detrimental association. Most associations were stronger for the AHEI, EDII and MIND and some in females and the oldest old. Although results were consistent for total, cardiovascular and neuropsychiatric chronic diseases, no associations were found with musculoskeletal multimorbidity. The study findings highlight the potential role of diet in the prevention of multimorbidity expansion in older populations, with possible implications for dietary guidelines, other public health strategies and clinical practice.

## Methods

### Study design and participants

The SNAC-K^55^ is an ongoing longitudinal community-based cohort of randomly sampled older adults aged ≥60 years in Stockholm, Sweden. The cohort was established in 2001–2004 (that is, wave 1), and participants are followed-up every 6 years, except for those aged ≥78 years who are followed up every 3 years (that is, waves 2, 3, 4, 5 and 6, conducted in 2004–2007, 2007–2010, 2010–2013, 2013–2016 and 2016–2019, respectively). Sociodemographic and clinical information is obtained through structured interviews and questionnaires conducted by trained physicians, nurses and psychologists as well as from medical records. In these analyses, we have included information on diet and potential confounders from waves 1–3 (that is, up to 6 years of follow-up; note that dietary data were not collected in waves 4–6) and information on multimorbidity from waves 1–6 (that is, up to 15 years of follow-up), corresponding to all available data for each variable.

From the 3,363 participants at baseline (73% response rate), we first excluded 877 who had inadequate information on diet (that is, ≥50% of answers missing in the food frequency questionnaire). We also excluded 13 participants who lacked information on potential sociodemographic confounders (seven on occupation, four on living arrangements and two on education). Participants without information on lifestyle-related potential confounders were assigned missing category indicators instead of being excluded, given the larger share of missingness. Accordingly, the analytical sample comprised 2,473 participants (note that no statistical methods were used to predetermine sample size). Data on attrition and mortality across the follow-up waves are presented in Extended Data Fig. 1.

SNAC-K was approved by the Regional Ethical Review Board in Stockholm, and written informed consent was obtained from the participants or their next of kin at each study visit. Participants were not compensated for taking part in the study.

### Dietary patterns

Dietary information was collected through a semiquantitative 98-item food frequency questionnaire^56^ with a nine-point Likert scale (ranging from ‘never’ to ‘four or more times a day’) in the first wave and a five-point scale in the second and third waves (ranging from ‘never or a few times per year’ to ‘two or more times a day’). Portion sizes were estimated with the help of color photographs, and daily energy and nutrient intakes were calculated using the food composition tables from the Swedish National Food Agency^56^. In this study, adherence to four existing dietary patterns was calculated: MIND^27^, AHEI^28^, AMED^29^ and EDII^30^.

The MIND is an a priori-defined score based on the foods and nutrients shown to be protective for dementia. Many of its dietary components are those of the Mediterranean and DASH diets, including emphasis on minimally processed plant-based foods and limited consumption of animal and high-saturated-fat foods^27^. The AHEI is based on a comprehensive review of the relevant literature and discussions with nutrition researchers to identify foods and nutrients that have been consistently associated with lower risk of chronic disease in clinical and epidemiological investigations^28^. The AMED is based on the MDS, which reflects adherence to the traditional Mediterranean diet^17^. It incorporates several modifications based on dietary patterns and eating behaviors that have been associated with lower risk of chronic disease in clinical and epidemiological studies^29^. The EDII assesses diet quality based on its inflammatory potential. It is a weighted sum of anti-inflammatory and pro-inflammatory food groups and has been found to predict concentrations of plasma inflammatory markers. Unlike the other three dietary patterns, higher adherence to EDII indicates a less healthy diet^30^. Information on the items of the patterns, scoring systems used and mean scores for every dietary pattern and food item can be found in Supplementary Table 8.

### Multimorbidity

Chronic diseases were assessed at every data collection wave by SNAC-K physicians, based on participant self-reports, medical charts, anamnestic details and information gathered from participants’ proxies. To increase the reliability of the diagnoses, additional clinical, laboratory and drug-related parameters complement physicians’ assessments. Inpatient and specialist outpatient diagnostic data from the Swedish National Patient Register were also included. Based on a consensus definition of chronic disease, all four-digit level codes from the International Classification of Diseases, 10th revision (ICD-10) were classified as chronic or not by an international and multidisciplinary team. Chronic ICD-10 codes were subsequently grouped into 60 broader categories according to clinical criteria, as described in a previous study (Supplementary Table 9)^57^. The prevalence of all chronic disease categories at baseline and end of follow-up is shown in Supplementary Table 10.

Multimorbidity was operationalized as the total number of chronic diseases and further grouped into three organ systems^8^: (1) cardiovascular diseases (ischemic heart disease, heart failure, atrial fibrillation, cerebrovascular disease, cardiac valve diseases, bradycardias or conduction diseases, peripheral vascular disease and other cardiovascular diseases); (2) neuropsychiatric diseases (depression and mood diseases, dementia, neurotic or stress-related and somatoform diseases, migraine and facial pain syndromes, peripheral neuropathy, Parkinson’s disease or parkinsonism, epilepsy, schizophrenia and delusional diseases, multiple sclerosis, other psychiatric or behavioural diseases and other neurological diseases); and (3) musculoskeletal diseases (dorsopathies, inflammatory arthropathies, osteoarthritis and other degenerative joint diseases, osteoporosis and other musculoskeletal and joint diseases).

### Potential confounders

**Extended Data Fig. 3:**
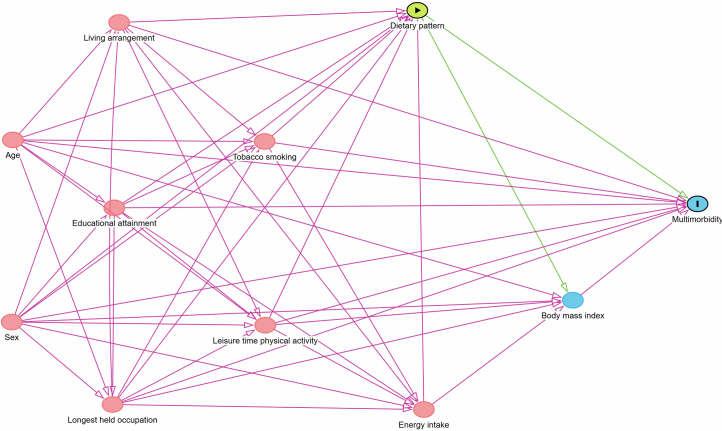
Directed acyclic graph describing the potential causal and confounding effects of diet quality on multimorbidity. Green arrows indicate a causal path between the exposure (green oval; **►**) and outcome of interest (final blue oval; **▌**). Pink arrows indicate biasing paths (that is, possible confounding factors [pink ovals] modifying the association between the exposure and the outcome of interest). Blue ovals preceding the outcome of interest indicate mediators.

Potential confounders of the study associations included age, sex, living arrangement (alone/not alone), longest-held occupation (manual/non-manual worker), educational attainment (elementary, high school or university), tobacco smoking (never, former, current or unknown), leisure time physical activity (inadequate, health-enhancing, fitness-enhancing or unknown)^58^ and energy intake (kcal d^−1^). A directed acyclic graph describing the potential causal and confounding effects of diet quality on multimorbidity is shown in Extended Data Fig. 3.

### Statistics and reproducibility

**Extended Data Fig. 4:**
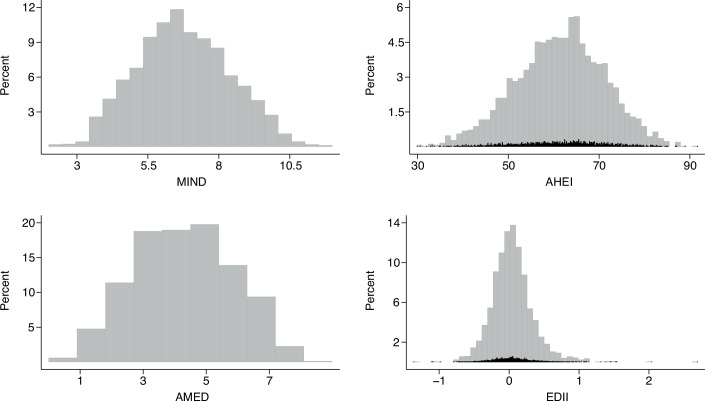
Baseline data distribution of the dietary patterns (N = 2,473). MIND: Mediterranean-DASH (Dietary Approaches to Stop Hypertension) Diet Intervention for Neurodegenerative Delay. AHEI: Alternative Health Eating Index. AMED: Alternative Mediterranean Diet. EDII: Empirical Dietary Inflammatory Index. Black spikes indicate individual data points. Note that the MIND and AMED are discrete in nature; therefore, individual data points match the bar charts.

First, we performed a descriptive analysis of the characteristics of the study population and their distribution across levels of adherence to the dietary patterns. Between-group comparisons relied on analysis of variance. In the main analyses, to assess the relationship between the adherence to each dietary pattern and the speed of chronic disease accumulation (that is, total and organ system specific), we used linear mixed models with random intercepts (participants) and random slopes (time from study inception). An interaction term between adherence to the corresponding dietary pattern (in 1-s.d. increments) and time from study inception (in years) was included as a fixed effect, indicating differences in the rate of chronic disease accumulation as diet quality increased (or decreased, in the case of the EDII). A negative interaction term (that is, β coefficient) indicated a less steep yearly accumulation of chronic diseases for every s.d. increase in the dietary pattern and vice-versa. The associations between adherence to the dietary patterns and the baseline number of chronic diseases (that is, fixed effect of diet quality on chronic diseases at time zero) are also shown. Models were adjusted for potential confounding variables, as follows: Model 1 included age and sex; Model 2 was additionally adjusted for living arrangement, occupation and education; and Model 3 was additionally adjusted for tobacco smoking, physical activity and energy intake. Models on disease accumulation within a given organ system were adjusted for diseases not belonging to said organ system. Data distribution of the dietary patterns was assumed to be normal, but this was not formally tested (Extended Data Fig. 4).

To reduce measurement error of diet and covariates, we used the cumulative average adherence to the dietary patterns (across waves 1–3), the cumulative average of continuous potential confounders (across waves 1–3) and the most recent information on categorical potential confounders (across waves 1–3) and comorbidities (across waves 1–5). For example, for a participant with dietary assessments available for waves 1, 2 and 3, diet quality at wave 1 was used for the first observation; the average diet quality between waves 1 and 2 was used for the second observation; and the average among waves 1, 2 and 3 was used for the third and successive observations.

We examined three-way interactions between every dietary pattern, time from inception and every potential confounder included in the models. Because several significant interactions with sex and age arose, study associations are also shown for males and females and for persons <78 years and ≥78 years of age separately. To account for potential false-positive results stemming from multiple testing, we calculated the 5% FDR for the 16 three-way interactions with sex and age (that is, four dietary patterns and four chronic disease models)^59^.

Numerous sensitivity analyses were conducted: (1) restricting the analyses to participants without multimorbidity at baseline (disregarding hypertension and dyslipidaemia, due to their high prevalence), to participants who were followed-up at least once and to observations with data on every potential confounder (complete case analysis); (2) using only baseline data for both diet and potential confounders; (3) excluding participants with probable cognitive impairment at baseline (Mini-Mental State Examination ≤24)^60^ and not updating dietary information when such condition presented in the follow-up; (4) excluding observations with likely misreported dietary data (>5,000 kcal d^−1^ or <800 kcal d^−1^ in males and >4,000 kcal d^−1^ or <500 kcal d^−1^ in females^61^); (5) adjusting the analyses for consumption of sugar-sweetened beverages (g d^−1^) when not included in the corresponding dietary pattern; (6) not considering potential cardiovascular risk factors (that is, hypertension, obesity and dyslipidemia), sleep disorders and genetic conditions (that is, chromosomal abnormalities) in the chronic disease count; (7) using the Royal College of Surgeons Charlson score instead of the total chronic disease count as the study outcome^62^ (adapted for the Swedish ICD-10 code system by the Swedish National Board of Health and Welfare and ranging from 0 to 29, with higher scores indicating higher comorbidity burden); and (8) assessing diet quality with the GDQS^63^ and the MDS^17^, as opposed to the AHEI and the AMED.

We also conducted additional analyses using group-based trajectory modeling, a complementary technique that gathers individuals into meaningful subgroups that show statistically similar trajectories over time for a given outcome^64^. To do so, we first identified age-adjusted and sex-adjusted disease accumulation trajectories for total, cardiovascular, neuropsychiatric and musculoskeletal chronic diseases, selecting the number of trajectories according to the best-fit model based on the Bayesian information criterion. Trajectories were subsequently included as the outcome variable in multinomial logistic regression models, adjusted for all potential confounders described above, with the aim of exploring the associations of adherence to the dietary patterns with every possible disease trajectory. Here, each participant was assigned to the most likely trajectory of multimorbidity accumulation speed based on the posterior probabilities of group membership obtained from the group-based trajectory model. Because said probabilities did not change over time, we used average data from the first three waves for both diet and continuous potential confounders as opposed to cumulative averages and baseline data for categorical potential confounders. Similarly to the main analyses, models on disease trajectories within a given organ system were adjusted for the trajectories of diseases not belonging to said organ system. The associations between dietary patterns and multimorbidity trajectories are presented as log-odds.

All analyses were performed using Stata 17 statistical software (StataCorp LLC).

### Reporting summary

Further information on research design is available in the Nature Portfolio Reporting Summary linked to this article.

## Supplementary information

Supplementary Tables 1–10

Reporting Summary

## Data Availability

SNAC-K data are sensitive data; thus, they cannot be shared publicly, but raw and analyzed deidentified data can be requested by qualified researchers at https://www.snac-k.se/. The request will be reviewed to ensure confidentiality and intellectual property obligations. A data-sharing agreement must be signed prior to data release.

## Code availability

No custom code or mathematical algorithm was employed in this study. Analytical scripts for this study are available from the corresponding authors upon reasonable request.
