# Supplementary material for: Dietary patterns and accelerated multimorbidity in older adults
Source: Nat Aging. 2025 Jul 28;5(8):1481–90. doi: 10.1038/s43587-025-00929-8 (PMC12350159; doi:10.1038/s43587-025-00929-8)
Supplement: Supplementary file 2 — Reporting Summary [file 43587_2025_929_MOESM2_ESM.pdf]

nature portfolio

Corresponding author(s): David Abbad-Gomez, Adrián Carballo-Casla

Last updated by author(s): 24/05/2025

Reporting Summary

Nature Portfolio wishes to improve the reproducibility of the work that we publish. This form provides structure for consistency and transparency in reporting. For further information on Nature Portfolio policies, see our Editorial Policies and the Editorial Policy Checklist.

Statistics

For all statistical analyses, confirm that the following items are present in the figure legend, table legend, main text, or Methods section.

n/a	Confirmed	
☐	☒	The exact sample size (*n*) for each experimental group/condition, given as a discrete number and unit of measurement
☐	☒	A statement on whether measurements were taken from distinct samples or whether the same sample was measured repeatedly
☐	☒	The statistical test(s) used AND whether they are one- or two-sided<br>*Only common tests should be described solely by name; describe more complex techniques in the Methods section.*
☐	☒	A description of all covariates tested
☐	☒	A description of any assumptions or corrections, such as tests of normality and adjustment for multiple comparisons
☐	☒	A full description of the statistical parameters including central tendency (e.g. means) or other basic estimates (e.g. regression coefficient) AND variation (e.g. standard deviation) or associated estimates of uncertainty (e.g. confidence intervals)
☐	☒	For null hypothesis testing, the test statistic (e.g. *F*, *t*, *r*) with confidence intervals, effect sizes, degrees of freedom and *P* value noted<br>*Give P values as exact values whenever suitable.*
☒	☐	For Bayesian analysis, information on the choice of priors and Markov chain Monte Carlo settings
☐	☒	For hierarchical and complex designs, identification of the appropriate level for tests and full reporting of outcomes
☒	☐	Estimates of effect sizes (e.g. Cohen's *d*, Pearson's *r*), indicating how they were calculated

Our web collection on statistics for biologists contains articles on many of the points above.

Software and code

Policy information about availability of computer code

Data collection	No special software was used for data collection. Data was obtained through questionnaires, interviews, and linkage to existing Swedish administrative and health-related databases.
Data analysis	All analyses were performed using the statistical software STATA 17 (StataCorp LLC, USA). For group-based trajectory analyses, we used the STATA package TRAJ, version 9.4.

For manuscripts utilizing custom algorithms or software that are central to the research but not yet described in published literature, software must be made available to editors and reviewers. We strongly encourage code deposition in a community repository (e.g. GitHub). See the Nature Portfolio guidelines for submitting code & software for further information.

Data

Policy information about availability of data

All manuscripts must include a data availability statement. This statement should provide the following information, where applicable:

- Accession codes, unique identifiers, or web links for publicly available datasets
- A description of any restrictions on data availability
- For clinical datasets or third party data, please ensure that the statement adheres to our policy

SNAC-K data (including linked data from the Swedish National Patient Register) are sensitive data; thus, they cannot be shared publicly, but raw and analyzed de-

identified data can be requested by qualified researchers at https://www.snac-k.se/. The request will be reviewed to ensure confidentiality and intellectual-property obligations. A data-sharing agreement must be signed prior to data release.

Research involving human participants, their data, or biological material

Policy information about studies with human participants or human data. See also policy information about sex, gender (identity/presentation), and sexual orientation and race, ethnicity and racism.

Reporting on sex and gender	Information concerning sex (a biological attribute) is available in the SNAC-K dataset, whereas that on gender is not. Analyses have been stratified by sex and reported in one of the main figures.
Reporting on race, ethnicity, or other socially relevant groupings	Given that the study population comprised older adults from an affluent neighborhood in Stockholm, Sweden, race and ethnicity information was not collected at the study onset.<br>Other socially relevant groupings included living arrangement (alone/not alone), longest held occupation (manual/non-manual worker), and educational attainment (elementary, high school, or university).
Population characteristics	Mean age at baseline was 71.5 years (standard deviation [SD] 9.39), 61.1% of the study participants were females, and 84.3% had multimorbidity. The majority of participants were followed at least once or twice (80.1% and 63.8%, respectively) over the 15-year study period.
Recruitment	The study population consists of adults ≥60 years living in the community or in institutions, from the Kungsholmen district of Stockholm, Sweden. Before the visit, the participant was invited by a letter or a phone call and informed by a detailed letter about the purpose of the study and the duration and interview process. A random sample of 11 age cohorts born between 1892 and 1939 (the youngest and oldest age cohorts were oversampled) was invited to participate in the study. People who agreed to participate were evaluated for the first time between 2001 and 2004. Participants who were <78 years of age were then followed up every six years and participants ≥78 years every three years. The present study is based on data collected at baseline, three years, six years, nine years, twelve years and fifteen years. At baseline, 3363 people were examined (participation rate 73%). Non-participants were older, more likely females, and more likely institutionalized. As a consequence, non-participants may have presented a higher disease burden. A participation rate of 73% remains among the highest reported in population-based studies involving older people.
Ethics oversight	SNAC-K was approved by the Regional Ethical Review Board in Stockholm, and written informed consent was obtained from the participants or their next of kin at each study visit. Participants were not compensated for taking part in the study.

Note that full information on the approval of the study protocol must also be provided in the manuscript.

Field-specific reporting

Please select the one below that is the best fit for your research. If you are not sure, read the appropriate sections before making your selection.

☒ Life sciences ☐ Behavioural & social sciences ☐ Ecological, evolutionary & environmental sciences

For a reference copy of the document with all sections, see nature.com/documents/nr-reporting-summary-flat.pdf

Life sciences study design

All studies must disclose on these points even when the disclosure is negative.

Sample size	No sample-size calculation was performed. All available participants from the original cohort (after managing exclusions) were included.
Data exclusions	From the 3363 participants at baseline (73% response rate), we first excluded 877 who had inadequate information on diet (i.e., ≥50% of answers missing in the food frequency questionnaire [FFQ]). We also excluded 13 participants lacking information on potential sociodemographic confounders (seven on occupation, four on living arrangements, and two on education). Participants without information on lifestyle-related potential confounders were assigned missing category indicators, given the larger missingness. Accordingly, the analytical sample comprised 2473 participants.
Replication	Study results were similar in minimally-adjusted and fully-adjusted models, and replicated in several sensitivity analyses. Data is available for qualified researchers to replicate the study findings (see data availability statement).
Randomization	There was no random allocation of participants, as this is an observational study.
Blinding	Blinding was not relevant for this is observational study. The chronic disease diagnoses were performed years before the statistical analyses.

Reporting for specific materials, systems and methods

We require information from authors about some types of materials, experimental systems and methods used in many studies. Here, indicate whether each material, system or method listed is relevant to your study. If you are not sure if a list item applies to your research, read the appropriate section before selecting a response.

Materials & experimental systems

n/a	Involved in the study
☒	☐ Antibodies
☒	☐ Eukaryotic cell lines
☒	☐ Palaeontology and archaeology
☒	☐ Animals and other organisms
☒	☐ Clinical data
☒	☐ Dual use research of concern
☒	☐ Plants

Methods

n/a	Involved in the study
☒	☐ ChIP-seq
☒	☐ Flow cytometry
☒	☐ MRI-based neuroimaging

Plants

Seed stocks	Not applicable
Novel plant genotypes	Not applicable
Authentication	Not applicable
